# Editorial: Advancing social equity through microbiology

**DOI:** 10.3389/fcimb.2023.1260284

**Published:** 2023-08-02

**Authors:** Laura M. A. Oliveira, Ariangela Kozik, Kathryn Milligan-McClellan, Ada K. Hagan

**Affiliations:** ^1^Departamento de Microbiologia Médica, Instituto de Microbiologia Paulo de Góes, Universidade Federal do Rio de Janeiro, Rio de Janeiro, RJ, Brazil; ^2^Department of Molecular, Cellular, and Developmental Biology, University of Michigan, Ann Arbor, MI, United States; ^3^Department of Molecular and Cell Biology, University of Connecticut, Storrs, CT, United States; ^4^Alliance SciComm & Consulting, LLC, Russellville, TN, United States

**Keywords:** innate immunity, social equity, research frameworks, clinical microbiology, microorganism

Efforts have been made by academic institutions and scientific societies to assure that a diverse and inclusive community of scientists have a voice in Microbiology research. However, given a history of exclusion in the sciences, scientists from low-and-middle income countries (LMICs) and those from historically marginalized groups in North America (e.g. women, Black, Indigenous, Latinx, and LGBTQIA+ people) have struggled to have their voices and interests heard (Ishaq et al., 2019, 2021). Microbiological topics that are extremely important to poor populations living in Africa and South America, for example neglected infectious diseases like leprosy, are not highlighted nor recognized as high-priority scientific topics to be investigated and/or funded (Houweling et al., 2016). This leads to a vicious cycle in which poor and underprivileged people don’t reap the benefits of scientific advancements that may improve their quality of life because their needs and perspectives have been overlooked.

Microbes are everywhere, including our bodies, animals, and the environment, and they play a key role in human health. Microorganisms do not distinguish between privileged and marginalized groups of people, but lower socioeconomic status and/or less access to resources combined with the lack of access to proper health care can lead to unfavorable interactions with microorganisms that can culminate in disease. In this way, microbiology is intimately related to social equity (Ishaq et al., 2019; Farmer et al., 2021; Ishaq et al., 2021). For example, the gut microbiota reflects the type of diet we consume every day. Evidence suggests that a lack of access to nutrient-rich food and a diet based on low-food diversity (as is common for people and places impacted by poverty) can jeopardize the formation of a healthy gut microbiome and increase the risk of obesity, diabetes, anxiety and depression, and cardiovascular diseases (Wilson et al., 2020). Consider also that the highest burden of neglected infectious diseases, like leprosy, Chagas’ disease, and schistosomiasis, are reported in LMICs. Poor people from LMICs lack access to health care and basic sanitation and live in crowded areas that facilitate the transmission of microorganisms (Houweling et al., 2016). In addition, empirical misdiagnosis is often an issue in this context due to the unavailability or lack of cost-effective diagnostic methods that allow an early, accurate diagnosis. Equitable access to improved diagnostic methods can provide a more efficient control of neglected infectious diseases, leading to adequate treatment and better patient outcomes (Ling et al., 2019; Bharadwaj et al., 2021). Social inequity also appears in the development of preventative therapies like vaccines. Certain infectious diseases affect people worldwide, like neonatal meningitis caused by Group B *Streptococcus*, but most of the data that serve as the basis for the development of global prevention strategies, especially vaccines, come from high-income countries (HICs). The resultant vaccines, however, may have low efficacy in LMICs as their dominant strains were not accounted for or included during development (World Health Organization (WHO), 2021, Ali et al., 2022). Equity in microbiology demands that studies to characterize pathogens that cause severe human infectious diseases must be conducted in LIMCs as well as HICs to inform vaccine design and ensure the universal coverage of future vaccines.

The goal of this Research Topic, *Advancing social equity through microbiology*, was to expand the scope of Frontiers and as a result highlight historically marginalized voices in cellular and infection microbiology as a way to contribute to advance understanding of the reciprocal impacts of social equity and Microbiology.

Our Research Topic includes five articles that cover topics and/or populations relevant to the issues described above. Ji et al. investigated the vaginal microbial composition and the pregnancy outcome of frozen embryo transfer (FET) for a group of Chinese women. This work showed that a Lactobacilli-dominant vaginal microbiota was a favorable factor for a positive clinical outcome of FET, while asymptomatic bacterial vaginosis negatively correlated with FET outcomes. Tang et al. used a systems biology approach to analyze the bronchial bacterial microbiota of infants with recurrent wheezing and with or without atopic diseases to determine the pathogenesis of atopic wheezing and identify potentially diagnostic biomarkers. Wells et al. evaluated the role of gut bacteria in inflammation-related diseases of health disparities, including type 2 diabetes mellitus (T2DM) and obesity. Their community-based research approach recruited a cohort of Native Hawaiians and Pacific Islanders, a risk group for T2DM. Ramírez-Córdova et al. evaluated the clinical performance of a simple and cheap SARS-CoV-2 detection protocol based on a fast and intense sample homogenization followed by direct RT-qPCR. Morales-Jadan et al. published an opinion article describing the challenges faced and lessons learned during the COVID-19 pandemic, focusing on the quality of commercial SARS-CoV-2 nucleic acid tests available in the LMIC Ecuador.

We are grateful to Frontiers for providing us this opportunity to highlight this need in microbiology as well as to the authors who contributed to this Research Topic. We hope that other microbiology and immunology journals will similarly begin to focus on and recruit papers that highlight the intersections of microbiology and immunology with historically marginalized groups beyond microbiome research.

## Author contributions

LO: Conceptualization, Data curation, Methodology, Writing – original draft, Writing – review & editing. AK: Conceptualization, Methodology, Writing – review & editing, Data curation. KM-M: Conceptualization, Data curation, Methodology, Writing – review & editing, AH: Conceptualization, Methodology, Supervision, Writing – review & editing.
